# Supplementary material for: CYP6B Subtype Expression Fluctuates in the Great Mormon, Papilio memnon, with Changes in the Components of the Host Plants
Source: Insects. 2025 Feb 4;16(2):159. doi: 10.3390/insects16020159 (PMC11856695; doi:10.3390/insects16020159)
Supplement: Supplementary file 1 [file insects-16-00159-s001.zip › insects-3421000-supplementary.pdf]

Figure S1

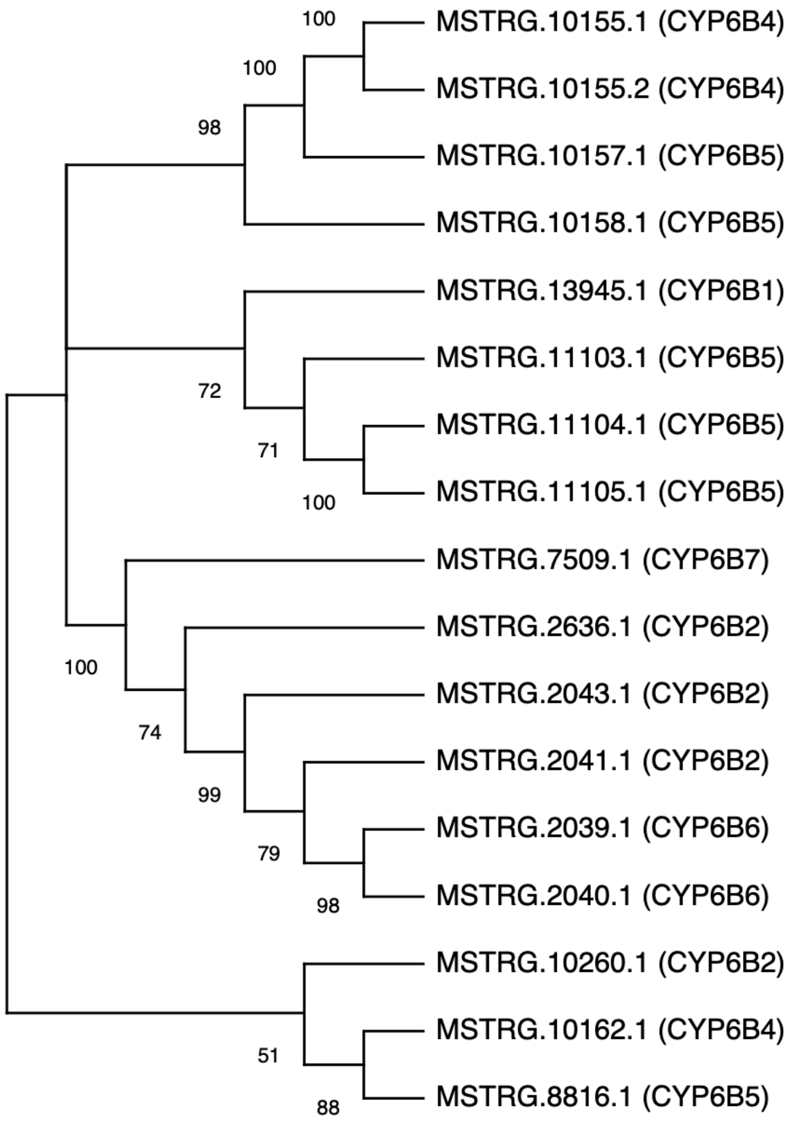

100
100
98
72
71
100
100
74
99
79
98
51
88
MSTRG.10155.1 (CYP6B4)
MSTRG.10155.2 (CYP6B4)
MSTRG.10157.1 (CYP6B5)
MSTRG.10158.1 (CYP6B5)
MSTRG.13945.1 (CYP6B1)
MSTRG.11103.1 (CYP6B5)
MSTRG.11104.1 (CYP6B5)
MSTRG.11105.1 (CYP6B5)
MSTRG.7509.1 (CYP6B7)
MSTRG.2636.1 (CYP6B2)
MSTRG.2043.1 (CYP6B2)
MSTRG.2041.1 (CYP6B2)
MSTRG.2039.1 (CYP6B6)
MSTRG.2040.1 (CYP6B6)
MSTRG.10260.1 (CYP6B2)
MSTRG.10162.1 (CYP6B4)
MSTRG.8816.1 (CYP6B5)

Figure S2

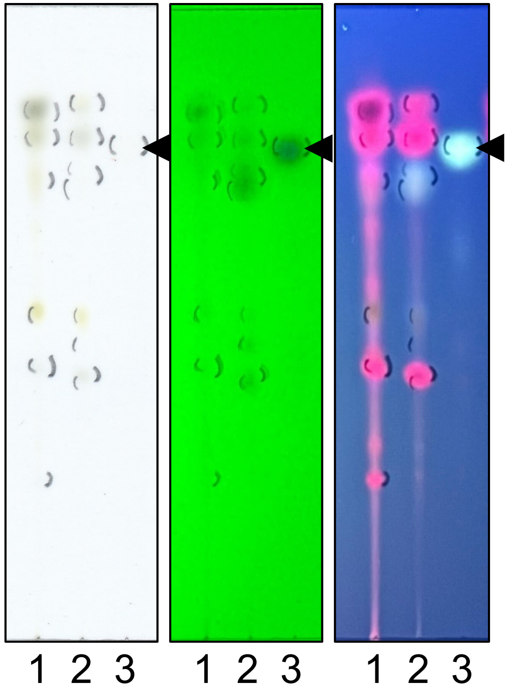

(a) (b) (c)

Table S1

Pm_transcript_IDs	Px_transcript_IDs	pident	length	mismatch	gapopen	qstart	qend	sstart	send	evalue	bitscore	Px_Gene_Name
MSTRG.13945.1	XM_013316294.1	82.88	1472	246	6	112	1580	69	1537	0	1317	PREDICTED: Papilio xuthus cytochrome P450 6B1 (LOC106120835), mRNA
MSTRG.2041.1	XM_013306334.1	79.038	1560	309	16	1493	3043	6	1556	0	1053	PREDICTED: Papilio xuthus cytochrome P450 6B2-like (LOC106113517), mRNA
MSTRG.2043.1	XM_013306337.1	84.375	1504	231	4	19	1520	195	1696	0	1472	PREDICTED: Papilio xuthus cytochrome P450 6B2-like (LOC106113520), mRNA
MSTRG.2636.1	XM_013316774.1	88.351	1571	178	3	3	1572	59	1625	0	1882	PREDICTED: Papilio xuthus cytochrome P450 6B2-like (LOC106121230), mRNA
MSTRG.10260.1	XM_013318651.1	84.843	1524	229	2	187	1709	27	1549	0	1533	PREDICTED: Papilio xuthus cytochrome P450 6B2-like (LOC106122588), mRNA
MSTRG.10155.1	XM_013314216.1	86.349	1509	202	4	9	1515	6	1512	0	1642	PREDICTED: Papilio xuthus cytochrome P450 6B4-like (LOC106119301), mRNA
MSTRG.10155.2	XM_013314216.1	86.349	1509	202	4	9	1515	6	1512	0	1642	PREDICTED: Papilio xuthus cytochrome P450 6B4-like (LOC106119301), mRNA
MSTRG.10162.1	XM_013314221.1	85.076	1521	227	0	63	1583	160	1680	0	1552	PREDICTED: Papilio xuthus cytochrome P450 6B4-like (LOC106119305), mRNA
MSTRG.8816.1	XM_013323145.1	86.148	1516	206	4	23	1536	56	1569	0	1633	PREDICTED: Papilio xuthus cytochrome P450 6B5-like (LOC106125793), mRNA
MSTRG.10157.1	XM_013314217.1	85.066	1520	225	2	1	1519	15	1533	0	1548	PREDICTED: Papilio xuthus cytochrome P450 6B5-like (LOC106119302), mRNA
MSTRG.10158.1	XM_013314222.1	83.645	1553	248	5	53	1602	13	1562	0	1456	PREDICTED: Papilio xuthus cytochrome P450 6B5-like (LOC106119306), mRNA
MSTRG.11103.1	XM_013318717.1	83.04	1533	254	6	20	1549	40	1569	0	1386	PREDICTED: Papilio xuthus cytochrome P450 6B5-like (LOC106122628), mRNA
MSTRG.11104.1	XM_013318667.1	82.804	1448	246	3	3	1449	122	1567	0	1291	PREDICTED: Papilio xuthus cytochrome P450 6B5-like (LOC106122599), mRNA
MSTRG.11105.1	XM_013318667.1	83.192	1529	240	14	1	1521	34	1553	0	1384	PREDICTED: Papilio xuthus cytochrome P450 6B5-like (LOC106122599), mRNA
MSTRG.2039.1	XM_013306304.1	80.743	1480	277	7	542	2017	171	1646	0	1147	PREDICTED: Papilio xuthus cytochrome P450 6B6-like (LOC106113499), mRNA
MSTRG.2040.1	XM_013306304.1	87.733	1663	197	7	356	2014	10	1669	0	1934	PREDICTED: Papilio xuthus cytochrome P450 6B6-like (LOC106113499), mRNA
MSTRG.7509.1	XM_013319474.1	86.426	1525	203	4	31	1553	1	1523	0	1666	PREDICTED: Papilio xuthus cytochrome P450 6B7-like (LOC106123243), mRNA

Table S2

Pm_transcript_IDs	a.value	m.value	p.value	q.value	rank	EstimatedDEG
MSTRG.10155.1	14.55692991	3.38174782	0.050030407	0.097206944	9414	0
MSTRG.10155.2	13.8571882	0.061298274	0.943135432	0.960249941	17965	0
MSTRG.10157.1	8.83491637	6.671525108	0.000518119	0.003126662	3031	1
MSTRG.10158.1	10.19448507	-7.285361396	0.00276014	0.010133986	4981	0
MSTRG.13945.1	9.145188447	-9.549856038	0.004018002	0.013416077	5478	0
MSTRG.11103.1	7.379043668	-6.794615006	0.004386549	0.014376343	5581	0
MSTRG.11104.1	7.878384012	-7.470773387	0.001011716	0.004991985	3707	1
MSTRG.11105.1	6.566065343	-3.839536997	0.071409467	0.128811693	10140	0
MSTRG.7509.1	-2.691041474	-9.130365741	0.098055242	0.16530216	10850	0
MSTRG.2636.1	13.67043954	3.929203706	1.92E-06	0.000234178	148	1
MSTRG.2043.1	8.154600032	7.277479803	6.92E-06	0.000319923	395	1
MSTRG.2041.1	7.638861589	10.98131777	0.000172961	0.001531281	2066	1
MSTRG.2039.1	9.891431558	4.774050889	0.000124738	0.001241337	1838	1
MSTRG.2040.1	16.26147444	3.236106035	0.001477175	0.006489713	4163	1
MSTRG.10260.1	10.21091236	-1.274771596	0.370095352	0.474250625	14273	0
MSTRG.10162.1	11.51079034	-0.887474854	0.001122942	0.005351676	3838	1
MSTRG.8816.1	13.77894498	7.160238691	7.19E-06	0.000323347	407	1

Table S3

Gene name	Forward primer (5'–3')	Reverse primer (5'–3')
CYP6B2	GAGGGCTTGCGTTGGTGAAA	ACTACATTTGGCCGGTTCCA
CYP6B5	GCGGTTTGCGAAGGTTCAAT	CTTGGGGAAGAGAACGAGCC
CYP6B6	GCGTCCCCTTTTTACCATGC	GACAAAAGGGGTCGCCAAAG
rpL31	GTCGCGTAGACGCAATGATG	TTGATTGAGGCGACTGGCAC

>CYP6B2（MSTRG．2636．1）

TCCTGCTCCGCTAAAGAGCACACGTTACGACGCCAGTGTACGTTTCAAGTTAATATGTGGTATTATGTGT
TAGCATTTGTGATTGTGTTATATCTGTATAGTATCCGTGCATTTAACTATTGGAAGAAAAGAGGTATAAA
ACATGATCCTCCGATACCATTTCTTGGGAATAACATGCGACAGTTTTTCAAAAAGCAAGTATGGCTATG
TTAGCCACAGAAGCATACAAAAAATATCCAAATGACAAAGTAGTTGGATTCTTTAGAGGCTTGAAACCAG
AATTGGTAATAAGAGATCCAGCAATAGCGAAGAGAATTCTCGTTACTGATTTCCAACATTTTTATGCCAG
AGGTTTCAATCCTCATAAGACTGTTATAGAGCCACTTTTAAAAAACCTATTCTTTGCCGATGGTGACTTG
TGGCGTTTAATAAGGCAACGATTTACTCCGGCCTTTAGTACCGCTAAATTGAAAGGTATGTTTCCTATTA
TTACTGACCGAGCAGAAAAGTTACAAATCATCACAGAGGAAGTGTCTCATTTAGATTCATATGATGTTCG
CGAATTAATGGCAAGATATACTACTGATTTTATCGGCGCTTGCGGTTTCGGAATCTCCATGGACTCTTTG
AGCAATGAAAATTCTGAATTTAGAAGATTGGGTAAAAGAATTTTCCAACGAACCCCTAAAGATGCTATGT
TTGCGGCTCTGAAATTATTATTTCCGGAGTTATGTAAGAATCTTAATTTCTTAGATCCAGAATTAGAAAA
ATCCATGACTTTCTTGGTTCAAACTGTGATGAAAGAAAGAAATTATAAGCCCTCGGGGAAAAACGATTTT
ATCGATCTCATGTTAGAACTTAAAGAAAAAGGTACAATAATTGGCGAATCTATCGAGAGTAAAAATGATG
ACGGTACTCCAAAAGTAGTTACATTGGAAATGACAGACATGATGATGATAGCTCAAGTCTTTGTATTCTT
CGGAGCCGGATTCGAGACGTCATCTACAGCATCCAGTTACACTTTGCATCAATTAGCATTCAACCCAGAA
TATCAATTAAAAGTCCAAGAGGAAATTGATCAAGTCTTAAAAAAATACGATAATAAAATTACTTATGATG
CGGTAAATGAAATGACATATCTAGAAAAAGCTTTTTACGAAGCTATGAGAATGTACCCATCGGTAGCATA
CATTGTGAGAATGTGTACATCACCAAAATACACGATACCTGAAATTGGAGTTACAATAAACGAAGGTGTG
AAAGTTATGATCCCAATCCAAGCTATGCACAATGATGAAAGGTACTTTGAAGAACCAACAAAGTTCAACC
CCGAAAGATTTAATTTGGGTAGAAAACAATTTAGGGATGTGTTTTTACCTTTCGGCGAAGGTCCGAGGGC
TTGCGTTGGTGAAAGACTAGGACAGATGCAGTCTATGGCAGGTCTTGCAGCAGTTTTGCAGAAGTTTACG
GTGGAACCGGCCAAATGTAGTGTAAGGGATCCAAAACCGGAACCTACAGCAATAGTTGCAGAGGGCTTTG
TAGGCGGATTACCTCTTAAAATAAGGAAGAGAG

>CYP6B5（MSTRG．10157．1）

TAATTGTGTACAGTGCCGCAATGTTATACCTTTTAGTGACTTTGTCAAGTGTAGTTGCACTTTTATATTT
TTATTTTACAAGGACCTTTAACTACTGGAAGGATAGAAATGTACCTGGACCTAAACCATTACCATTCTTT
GGCAACCTTAAGGAAACCACTCTGAGACGTAAGCATGCTGCCGTAGTTTTTAAAAGTATTTACGATGCAT
ATCCCAATGAGAAAGTGGTTGGAGTTTACAGAATGACCACACCATGCCTATTGCTCCGCGACTTGGACGT
TATTAAGCACGTCATGATTAAAGACTTCGATCTGTTCGTCGACAGAGGAGTGGAATTCAGTAAAGAAGGA
CTAGGACTTAACCTGTTTCATGCTGACGGTGATACATGGAAAGTGCTAAGGAACAGATTTACACCAGTTT
TTACCTCTGGTAAATTGAGGAACATGTTGTATCTCATGATTGAACGGGGTGATCACTTTGTTGATTATGT
TGAGAACCTTCGTGCAAAAAATTCAGAACAGCCTATACATGTTCTCGTTCAGAAATTTACAATGGCCACT
ATCTCCGCTTGTGCCTTTGGCTTGGACTTGGACGAAGATATGTATCAAGTATTGAATAAAATAGACAAAA
TGATTTTTAC

TGCAAATTACAGCAACGAGTTAGATATGATGTACCCTGGCATATTGAAAAAGTTTAATGGTTCAATATTT
CCTAAATACGTAACCACTTTCTTCGATAACCTCGCACAAACTGTCGTCAAACAAAGAGGTGGAATGCCAA
CAAATAGAAAGGACTTCATGGATTTAATATTGGCGTTGAGGCAACAGAAAACAATTGAAGGAACGAAGAA
AATGGACAATGAGAAGCTGAGAATAGTTGAACTGACTGATACCGTAATTGCTGCCCAGGCGTTTGTATTT
TACGCGGCTGGCTATGAGACCAGCGCCTCCACCATGACGTACTTATTCTACGAATTGGCGAAACATCCTG
AGATACAAGATAAAGTCATTGCAGAAATTGACGAAGTTGTTAAGCGGTACAACGGTGAAATAAGCTACGA
CTGTTTGAATGAAATGACTTATTTGCAGCAAGTGTTTGATGAAACACTACGGAAATATCCAATTATAGAT
CCTTTGCAACGTAATGCGCAAATGGACTACACAATCCCAGGCACTAATGTTACTGTCAAGAAAGGACAAA
CGGTACTCGTGAATCCTATGGGTATCCATTATGACCCTAAACACTATCCCAACCCAGAGAAGTTTGATCC
TGAACGGTTTAGTCCTGAAAATGAGAAAGACAGACATTCGTGTGCTTACATGCCATTTGGGACTGGACCC
AGGAATTGTATTGGTATGCGGTTTGCGAAGGTTCAATCCCGTGTGTGCGTAGTCAAGTTTCTGTCGAAGT
ACCGAGTGGAACCTTCGAGAAATACACCAAACGTGTTGGAGTACGACCCCATGCGGCTCGTTCTCTTCCC
CAAGGGAGGAATTCATTTGAATGTAATAAGTAGATAAATGAAATCTATGCTTCGACGGTCATGACAACTA
AAGTAT

>CYP6B6 (MSTRG.2040.1)
GTTAACTACCACTTAAATTCGACACCTACTCACAGTATGATTTCGGTATGCAATGTACAGTACCGGTTTT
TAGCGGTAATATCGCCCTGGGCAAGATCTCGGCGTCCCCTTTTTACCATGCTTCGTTGGGACACTTAACA
GGGTTATGTGCGAATGGCTGACTTTGGCGACCCCTTTTGTCCAGCGCCCTGGGCGGTCGCCCAACCGCCC
CCTACCAAAACACCGGCACTGGATACGAGTTTATAAACTTGATAAACTATTTAAACATTTTTTAGTCTTC
GCATATTCACATATCTTGATTCTCGCGACTGCAGACGACTGACCGATGTTTAACTTTGGAGATACTTATA
TTGTCAATAATAATTTTGTACTGTGTAAAATTAATTTTAAATTTGTTCGTTTGTTATCAATTTGTTATCA
GACTTTGGGCTATATAAGTTTACGTACTGTTTGTCTACTGCCAAACCTGGCGCAATGTTGACCCTGGCTG
TTGTATTCGTGCTGTGCGTTGCTGTGTACTTGTATAGTACAAGGACGTTCACGTATTGGGAGAAAAGAGG
AATCAAACATGACAAGCCTATACCATTTTTTGGCAACAATTCACGTATGTACTTATGGAAGAAAAGTATG
ACACAAATTGGCGTGGAAATGTACTGGAAATACCCGAAGGAAAAGGTCGTCGGATTTTATAGAGCGATGC
GTCCGGAACTTGTGTTGAGAGATCCAGAAATTATAAAACGCGTCCTCGTTTCAGACTTTGGACATTTTCA
CATGAGAGGTATCAATACTCACAAAACCGTTGTTGAGCCCATAGCAAAAAACCTTTTTCTTGCTGAAGGC
GATCTCTGGAAACTGTTGAGGCAGCGCATGACTCCCGCGTTCACCAGCGGTAAACTAAAGGCGATGTTCC
CTCTCATTGTGGAGCGTGCTGAGCGCATGCAGAATCATGCAATCAATGCACCAACGACTGGACGAGTCCT
GGACTCTCGGGAAATAATGGCGCGGTATACTACTGACTTCATCGGTGCTTGTGGGTTCGGCCTCGACATG
GATTCTCTCAGTGTAGAAAATTCCTCTTTTAGACAACTCGGTATCGATATTTTTAAATTTGAAATTAGTC
AAATTGTTAAACAAATGCTTAAGGAGATGTTTCCTGAAGCATGCAAACATTTTAGAATATTCGGCAAAAT
AGAAAATGACGCGTACGCCCTTGTAAAATCCATTCTCCACAAGAGAAATTACGAACCATGTGGCAGGAAC
GATTTCATCGATCTTCTTTTGGAATGTAAGAAAAAAGGAAAAATAGTTGGACAATCTATAGAAAAAATGA
AATCGAATGGTACACCTGAAGAAGTTTCTATGGAATTAACCGAAGAAATAATGATGGCTCAAATTGTGGT

CTTTTTCGCCGCGGGTTTCGAAACTTCATCTTCCTCCACGAGCTATACGCTTCATGAACTCGCATATAAT
CCTGATGAGCAACGAAAAGTGCAAGAAGAAATCGATAGAGTTTTAGCGAAATATAATAACAAGCTCTGCT
ATGAAGCAGTTTCTGAAATGACTTACTTACATTGCGCTTTCAAAGAAGGCATTCGCATGTTTCCATCGTT
GGGGCATTTGGTCAGGAGGTGTGCTCGCAAATACACTTTCCCCGACTTGGACCTCGCGATAGACGAAGGT
GTTTCACTATATATACCTGTGCAGGCGTTGCACATGGACCCGTTGTATTTCGATGAGCCAGAGCAATTTA
AGCCAGGTCGTTTCTTAACTGATTTCATCAACCCGATGACGAGAAACATATATTTACCATTTGGAGAAGG
TCCCCGTGCATGTATTGGTGAGAGACTCGGTCTGATGCAGTCCCTAGCGGGTTTGGCGGCCGTGCTGTCC
CGGTACTCGGTTGAACCCGCGCCGGAAACGCTTCGCCATCCCAGGGTTGATCCCACTTCTAATATAGTGC
AAAGTGTCATTGGCGGTTTGCCTTTGATGTTTAAGTTGAGAAGTAAGTAAGTATGGTACATTTCACTTAG
GTACTTATATAAGATTTGCAGTGTCTTAAATTGGTGAAACAGCTTGTCTAATTTGATCATATTGTAATAA
TTTAATCTGCTCATAAATACTCTATATTGCTTTATTAAATTTTAGATAATTTGGTTATTTGCGGCATAAG
TAAGTTAAGTTTTTATTGCTTCAATGAATAAATATTTTTGATGGAATTATTGTTTCGAATTA
